# Epidemiology of Hepatitis B Virus Infection in Bangladesh: Prevalence among General Population, Risk Groups and Genotype Distribution

**DOI:** 10.3390/genes9110541

**Published:** 2018-11-08

**Authors:** Md. Hassan uz-Zaman, Ayesha Rahman, Mahmuda Yasmin

**Affiliations:** 1 International Center for Diarrhoeal Disease Research, Mohakhali, Dhaka 1212, Bangladesh; md.hassan@icddrb.org; 2 Department of Microbiology, Jagannath University, 9-10 Chittaranjan Ave, Dhaka 1100, Bangladesh; ayesha.du15@gmail.com; 3 Department of Microbiology, University of Dhaka, Dhaka 1000, Bangladesh

**Keywords:** hepatitis B, Bangladesh, prevalence, vertical transmission, occult infection, genotypes

## Abstract

Despite a considerable body of published research on hepatitis B in Bangladesh, researchers continue to lament the lack of reliable information about hepatitis B virus (HBV) infection epidemiology. The present review aims to provide a comprehensive survey of the literature with particular focus on a number of epidemiological questions, as well as a commentary on the trends of hepatitis B research as it has taken place in Bangladesh. The key themes to emerge from this review are: first, beyond noting a declining trend, it is difficult to provide conclusive estimates about HBV prevalence in the general population of Bangladesh. The majority of the studies, even the ones conducted on apparently healthy populations, fail to be adequately representative for the reasons explored in the article. Secondly, HBV infection in Bangladesh is sharply stratified across sociodemographic lines, which speaks to the role of awareness and risk exposure in HBV prevalence. Third, more research on occult infection rates is required to estimate the extent of risk posed by the current blood donation screening program, which relies exclusively on hepatitis B surface antigen as a biomarker. The same considerations apply for the comparative importance of vertical versus horizontal transmission and prevalence among particular risk groups like healthcare workers with high occupational exposure. Finally, while recent studies do allow us, albeit with some ambiguity, to draw conclusions about distribution of HBV genotypes in Bangladesh, there needs to be an added emphasis on molecular epidemiology. It is hoped that the present review, the first of its kind in Bangladesh, will serve as an up-to-date summary of the course HBV epidemiology research in Bangladesh has taken thus far, as well as crucial gaps to address going forward.

## 1. Introduction

Bangladesh, together with the Indian sub-continent, is recognized as a country with moderate prevalence of hepatitis B [1]. Beyond this rather surface-level characterization, there remains considerable ambiguity regarding the epidemiology of hepatitis B virus (HBV) infection in the country. Lack of reliable epidemiological information has been cited as one of the key challenges to effective hepatitis B response in Bangladesh [2]. And yet, a substantial number of studies on HBV prevalence in Bangladesh has indeed been conducted since the early 1980’s. In this context, there is a need for a comprehensive analysis of the findings of these studies which would guide both future research and policy. The present review aims to address this gap.

In order to characterize the review with a sharp focus, we sought to confine ourselves to a few key questions regarding HBV prevalence in Bangladesh. First, has enough epidemiological data been generated to draw precise, quantitative estimates about the prevalence of HBV in Bangladesh? Second, what do the studies targeting particular population groups tell us about the specific modalities of HBV transmission- for example, the extent to which vertical transmission is important in the spread of HBV, or vulnerabilities of particular risk groups? Third, what do we know about the distribution of HBV genotypes in Bangladesh? Answers to these questions are often nebulous and require substantial qualification, which necessitates a commentary on the general trends characterizing HBV epidemiology research in Bangladesh in the last decades. Based on these trends, we also provide suggestions as to the direction of future HBV research in Bangladesh. The present article does not aim to be a systematic review. However, the analysis presented here is hoped to be comprehensive enough to serve as an update to current HBV epidemiology in Bangladesh.

## 2. Materials and Methods 

We chose not to conduct a systematic review on this topic for a number of reasons. First, we anticipated a substantial body of grey literature to be available on the topic, mostly in the form of unpublished graduate or undergraduate dissertations. Material of this sort would be inaccessible from the extant databases. Further, by adopting a more flexible research strategy than one adopted in a systematic review, we can provide customized modes of analysis, tailored to the topics being discussed. As the studies considered are too heterogeneous to merit a meaningful meta-analysis, such flexibility in a narrative synthesis with a comprehensive scope would prove eminently useful.

To make sure our review captured as many of the published studies as possible, we conducted a systematic search of the PubMed and Bangladesh Journals Online (BanglaJOL, Bangladesh Academy of Sciences, Dhaka, Bangladesh) databases with the search terms (“hepatitis B” AND Bangladesh), which yielded a total of 217 journal articles. Following de-duplication, we conducted relevance screening based on titles and abstracts. The inclusion criteria were: primary studies on prevalence of HBV biomarkers among otherwise healthy populations, and/or groups exposed to particular risk factors (e.g., injection drug users, healthcare providers, people with a higher risk of sexually transmitted diseases) or other groups of interest (e.g., blood donors, pregnant women). Studies done on the molecular epidemiology of HBV (investigating prevalence of particular genotypes) were also considered. Studies done on particularly niche risk groups (e.g., patients “suspected of hepatitis B,” fulminant hepatic failure), chronic carriers, or other studies on groups which would not inform the general trends of HBV prevalence among the population at large (or key risk groups) were excluded. The article pool was supplemented with additions from the reference lists of the selected papers. In cases where the papers were not open access, they were obtained via the services of the International Centre for Diarrheal Disease Research, Bangladesh (icddr,b, Dhaka, Bangladesh) library and in the rare cases where icddr,b library archives did not have the journal issues, we extracted general prevalence data and population information from the study abstracts. Ultimately, the analyses in this review were based on a total pool of 61 articles—33 on otherwise healthy population groups, 24 on risk groups or other groups of interest and 4 on genotypes. Descriptive statistical analyses were considered sufficient for the aims of the article.

## 3. Hepatitis B Virus in the General Population

**Figure 1:**
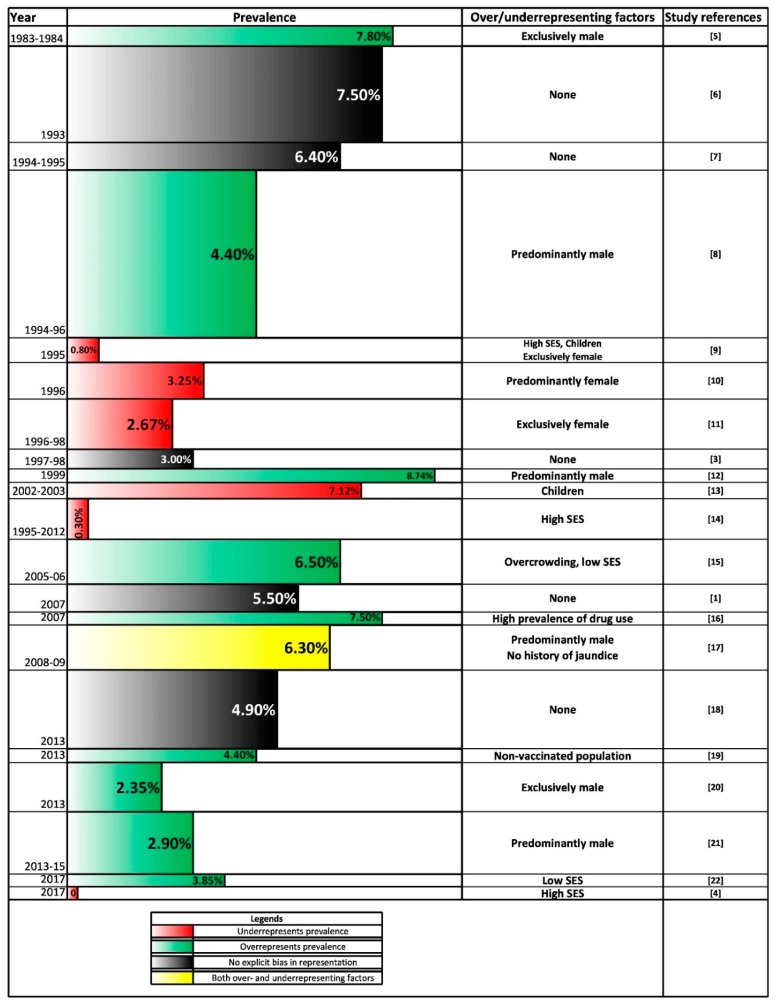
Studies reporting the prevalence of hepatitis B surface antigen among otherwise healthy populations in Bangladesh. The bar heights are factors of the normalized sample sizes of each study. The sample sizes ranged from 130 [3,4,5,6,7,8,9,10,11,12,13,14,15,16,17,18,19,20,21,22] to 43,312 [8]. Studies done on voluntary blood donors and pregnant women have not been included here, since they form a substantial enough group to merit their own discussion.

The expert review cited in the beginning of this article points to a lack of epidemiological data as far as the general population in Bangladesh is concerned [2]. This is primarily because most of the studies have investigated prevalence in particular risk groups and not the general population at large. These sentiments have been reflected by a number of authors [3,4]. While it is indeed accurate that a large number of studies have been done on particular risk groups, our survey yielded a substantial body of research, conducted from 1983 onwards, pertaining to study populations not explicitly characterized by any HBV risk factor. However, the results of these studies are highly heterogeneous and most of the study populations are not representative of the general population at large (Figure 1). The problem, as well as the general trends that could be gleaned from the study findings, are now discussed.

### 3.1. Choice of Biomarkers

**Figure 2:**
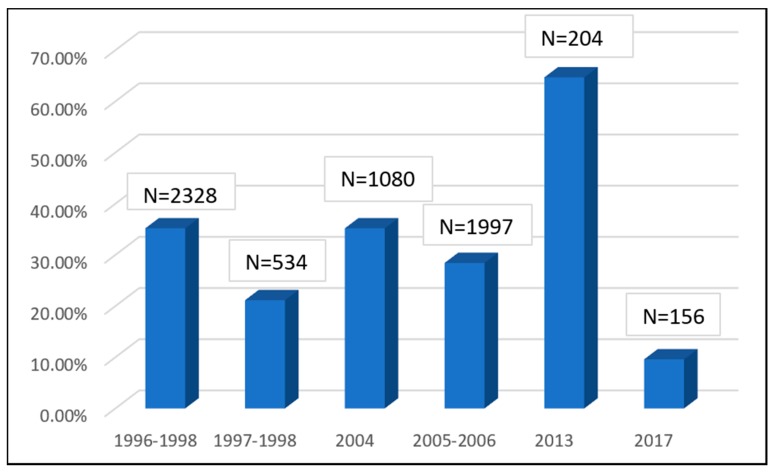
Prevalence of antibodies against hepatitis B core antigen (anti-HBc) among healthy populations in Bangladesh.

It is important to note at the outset that the majority of the studies under our consideration measure HBV prevalence exclusively via hepatitis B surface antigen (HBsAg). This biomarker detects acute and chronic infection but it is cleared from the body after the acute infection gets resolved. This is why measuring rates of HBsAg as well as antibodies against hepatitis B core antigen (anti-HBc) would be more informative, since the latter is retained in the body even after the infection is resolved. Only six studies reported the prevalence of anti-HBc among healthy population groups [3,4,11,15,19,23] and the range varies from around 10% to upwards of 60% (Figure 2). Considering how neither of the two studies documenting the highest [19] and lowest [4] prevalence represent the general population accurately (Figure 2), the actual range would probably be a lot tighter. For the remainder of our analysis, we will use the prevalence rates derived using HBsAg.

### 3.2. The Problem of Representation

As can be seen from Figure 1, almost all of the study populations are characterized by factors which are likely to contribute to either higher or lower rates of HBV prevalence compared to the general population in Bangladesh. For the majority of these studies, the design is post-hoc and study participants are selected based on convenience (e.g., as part of routine screening programs). For example, four studies [8,12,20,21] report HBV prevalence data collected during routine screening of overseas job seekers during their medical fitness examination. While such convenience sampling allows the availability of data based on large samples, the selected populations often have characteristics that do not align with those of the general population. In the present case, overseas job seekers in Bangladesh availing to that particular mode of processing are overwhelmingly male, a characteristic which also defines the four study populations. There are exceptions to this, for example Mahtab et al. [1] attempts to intentionally choose a sampling frame which would be representative of the general population of Bangladesh, as discussed later.

**Figure 3:**
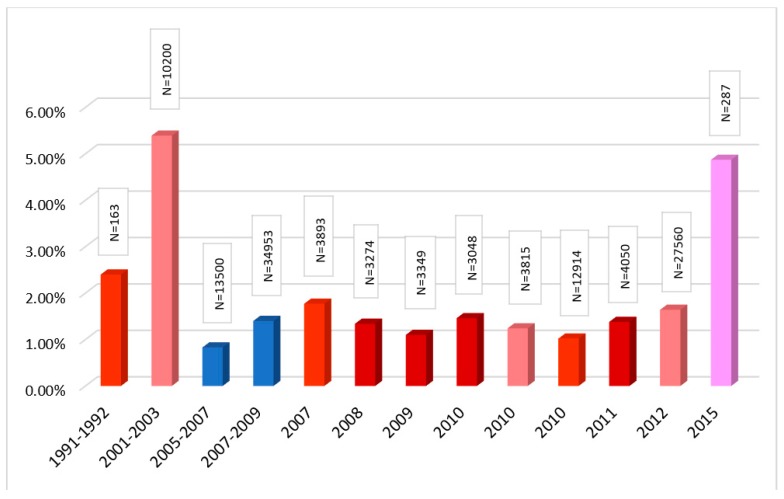
Hepatitis B surface antigen (HBsAg = prevalence among voluntary and replacement blood donors in Bangladesh. Darker hues of red indicate increasing male:female ratio in the study population (blue bars indicate no gender data was available). The percentage of males in the study population range from 81% [33] to 98% (2011 data in Reference [29]).

An extreme instantiation of this representation problem is given by the large number of surveys that has been conducted on blood donors. Each of these studies report the HBsAg prevalence data from routine screening of voluntary and party blood donors over a year or more. Like the studies done on overseas job seekers, most of these studies have very large sample sizes per year (with two exceptions, ranging from 3048 to 27,560). These surveys can be used as surrogate measures of HBV prevalence in the study region [24]. However, this extrapolation may be tenuous for a number of reasons. First, the voluntary blood donor population of Bangladesh tends to be predominantly male. The percentage of males in studies considered in this review range from 81% to 98% of the total study population (Figure 3). As discussed later in this paper, since HBV prevalence tends to be higher among males than females, the data from voluntary blood donor screening may overrepresent HBV prevalence in the general population. On the other hand, the voluntary blood donor population in a country may also be considered likely to possess a higher level of education, sense of social responsibility and awareness [1]. If accurate, studies based on this population would underrepresent the prevalence in the general population. Our analysis of the relevant published literature in Bangladesh [25,26,27,28,29,30,31,32,33] suggests this latter trend, since with three exceptions, reported prevalence among voluntary blood donors is confined between 0.83% to 1.77% (Figure 2). This range of values is lower than almost all of the prevalence data reported in otherwise healthy populations (Figure 1). These facts significantly limit the epidemiological utility of these studies conducted on voluntary blood donors.

The upshot of this discussion is that despite the existence of a substantial body of literature on HBV prevalence in otherwise healthy individuals in Bangladesh, most of their sampling frames do not represent the population at large. In effect, only five studies under our consideration lacked any explicit evidence of under- or over-representation [1,3,6,7,18].

### 3.3. Current Prevalence

Some researchers have pointed out that HBV prevalence in Bangladesh is at a decline [34]. As Figure 1 illustrates, reports of HBV prevalence are characterized by significant heterogeneity between them (0% to 8.74%), much of which can be attributed to the problem of representation discussed in the foregoing section. This heterogeneity, however, does not mask the fact that HBV prevalence is indeed currently lower than it used to be—0% to 4.9% in the past five years, compared to 7.8% in the early 1980’s. Also, if we focus on the studies conducted on larger sample sizes (>1000)—none of the studies from 2005 onwards, overrepresentation notwithstanding, report prevalence rates approaching 7% or higher.

Beyond this observation, the predominance of non-representative studies makes it difficult to tighten our estimations regarding current prevalence. However, there are important exceptions to this. The study conducted in 2007 was designed to appropriately represent the population in Bangladesh [1]. The study site was chosen to be an area near the outskirts of the capital city, which was frequented by people all over the country due to its industrial importance. Further, instead of attempting to piggy-back on a pre-existing screening program, a recruitment mechanism was constructed for the express purpose of HBsAg screening among the community, aided by religious and community leaders. The study population was considerably diverse in terms of gender, age and socio-economic background. The confluence of these factors makes the findings of this study—a 5.50% prevalence rate—fairly representative of the Bangladeshi population in general. Despite these merits, the study was conducted over a decade ago and is arguably less representative of the prevalence rate today. A later study conducted in 2013, based on people attending a hospital in Dhaka for routine medical check-up, reported a prevalence rate of 4.90% [18]. Studies conducted since then have consistently generated even lower rates of prevalence, notwithstanding overrepresentation due to demographic bias. This indicates that HBV in Bangladesh is indeed at a decline, even at finer time-scales and a cautious approximation would suggest that its prevalence is very likely in the neighborhood of 4% or lower.

### 3.4. Effects of Sociodemographic Variables

**Figure 4:**
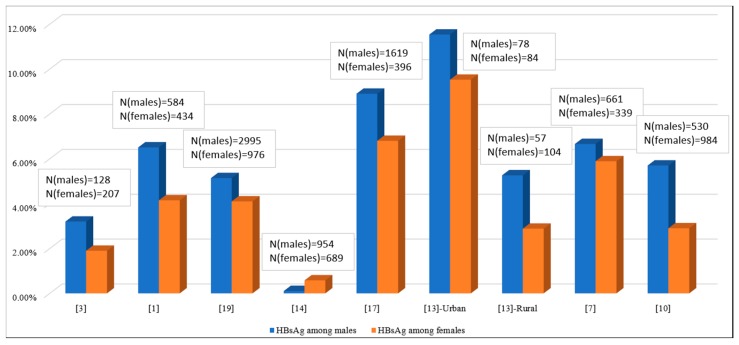
Comparative prevalence of HBsAg between males and females in study populations.

The fact that HBV prevalence tends to be higher among males than females has been recognized since the discovery of HBsAg (then Australia antigen) itself [35]. Almost all of the reports on HBV prevalence in Bangladesh which provide gender-specific data are in line with this fact (Figure 4) and in most cases the difference is statistically significant. More noteworthy perhaps is the prominent role played by socio-economic status. Among the studies considered, populations characterized by high socioeconomic status [4,9,14] had the lowest reported prevalence of HBV by far, ranging from 0% to 0.8% (Figure 1). All three of these studies surveyed children or young adults enrolled in a school or college in an urban area of a high socio-economic background. Their profile as young students in private schools and universities may also correlate with increased awareness and consequently lower involvement in behavior that contributes to disease risk. On the other hand, study populations with a lower socioeconomic status consistently showed higher prevalence. The study reporting a 6.50% rate is a case in point [15], as it was conducted in one of the most densely populated areas in urban Dhaka characterized by overcrowding and poor sanitation.

The effect of both of these demographic variables—gender and socioeconomic status—can be explained in terms of the differential exposure to risk factors. The study done in impoverished urban Dhaka, for example, report higher prevalence of certain risk factors associated with HBV in the study population—79% of the population visited unregistered healthcare providers and 69% of the males in the population underwent circumcision (the majority of which are likely to have been conducted by unregistered “doctors”) [15]. These rates are higher than what can be approximated as rates representing the general population in the time frame—61.39% and 59.63% for visiting unregistered healthcare providers and male circumcision, respectively [1]. Both of the studies have implicated these behaviors as being associated with HBV prevalence. Similar conclusions can also be drawn in the case of [16], a study conducted on garment workers of a low socio-economic background. The unusually high rate of prevalence can be attributed to the fact that of the 11 individuals who were positive for HBsAg, 8 had a history of injection drug use. These considerations cumulatively demonstrate that the disparity of HBV prevalence across sociodemographic groups can be partly and significantly, attributed to an increased risk of exposure to behaviors associated with high prevalence. 

## 4. Hepatitis B Virus Prevalence among Major Risk Groups

As has been pointed out by a number of authors [1,3,4,15], HBV epidemiological research in Bangladesh have focused on risk groups to a substantial extent (Table 1). Perhaps understandably, the risk group that has been discussed most often has been drug users. The studies cited often include data on both injection (IDU) and non-injection drug users (NIDU) but IDUs consistently have a higher prevalence of HBV. Citing the prevalence of drug users as a whole—both IDUs and NIDUs—often mask this inter-group disparity. In a study based on drug user statistics collected over a period of five years [36], HBsAg prevalence in IDU and NIDUs were 19.1% and 3.16%, respectively. This trend is seen in most of the studies, although [37] report lack of significance difference between the two groups. Also, studies done on the general prevalence of HBV in Bangladesh identify injudicious injection use as one of the key risk behaviors for the disease [1]. The most recent studies report a 7–7.5% prevalence among the injection drug users in Bangladesh.

The rest of the risk groups have only been the subject of two or three studies each. No recent data is available on the prevalence rate among commercial sex workers and studies done on thalassemic patients are presented as a surrogate for multitransfused patients. There are two studies, however, which investigated HBV prevalence among truck drivers and helpers [38] and women who live near truck stands [39], groups considered to be of a higher risk of contracting sexually transmitted infections due to their comparatively unsafe sex lives. Individuals in the trucking industry and women living near truck stands had prevalence rates of 5.9% and 3.6% respectively, which are rates comparable to the general prevalence among the population at the time.

The situation with healthcare workers, a particularly important risk group for HBV transmission, requires more comment. The prevalence rates presented in the table masks their inter-group disparity. For example, in the study done by Biswas et al. [50], the group consisting of doctors and nurses have a prevalence rate of 1.9%, while technicians and assistants have rates of 15% and 10%, respectively. The same conclusions can be drawn from [53], where the prevalence among doctors and nurses were 0.59% but an unspecified “auxiliary staff” group had a prevalence rate of 7.32%. [52] also reports a prevalence rate of 0% for physicians. There is considerable disparity between vaccination rates of doctors and technicians (100% vs. 45%). The discussion, therefore, points to a conjunction of disparities between occupational exposure and level of awareness as being responsible for the higher rates of HBV among healthcare personnel at risk.

## 5. Vertical Transmission of Hepatitis B Virus in Bangladesh

Vertical transmission of HBV, defined as “positivity at 6–12 months of life of the hepatitis B surface antigen (HBsAg) or of HBV-DNA in an infant born to an infected mother” [54], is said to be one of the most common modes of transmission in endemic areas. Vertical transmission of virus is important not only because of clinical consequences thereof—in excess of 90% of cases of vertically transmitted infections result in chronicity [55]—but also because of the complications that arise in management of the infection during pregnancy. Data on the significance of vertical transmission of HBV in Bangladesh, however, is ambivalent and the present section will explore the ambivalence.

**Figure 5:**
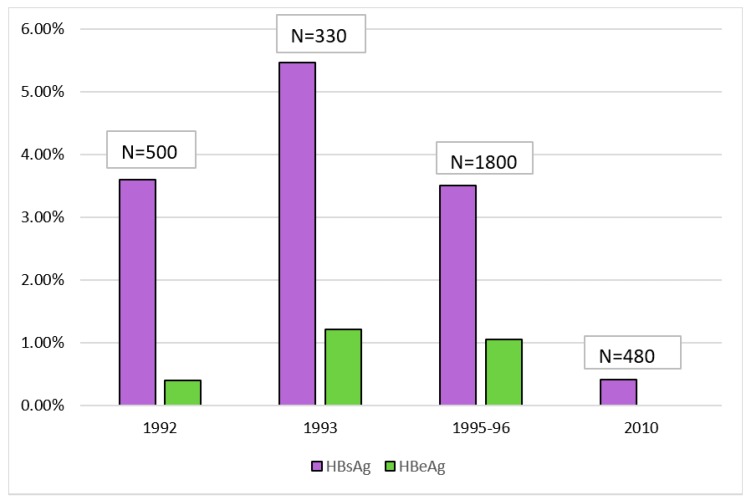
Prevalence of HBV biomarkers among pregnant women and new mothers in Bangladesh.

**Figure 6:**
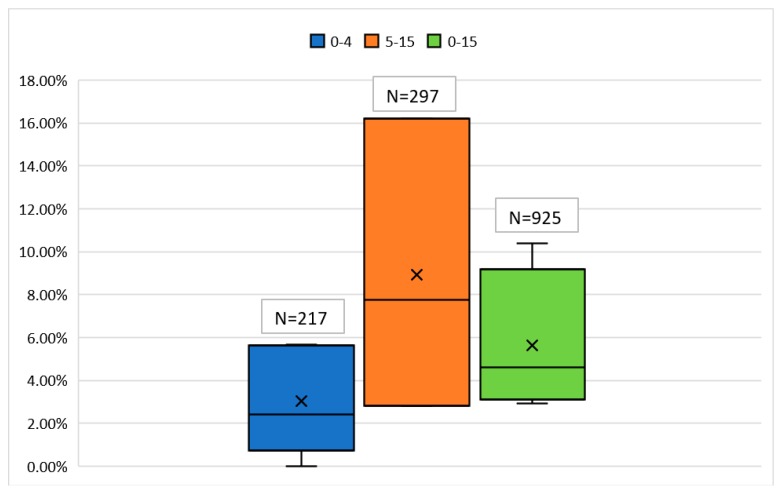
Comparative prevalence of HBsAg among under-15 Bangladeshi children of different age groups.

The first indicator of the degree of vertical transmission of HBV would be prevalence among the general female population. Extrapolating our earlier approximations (Figure 1), the prevalence among the general female population in Bangladesh is likely to be less than 4%. Higher risks of vertical transmission (up to 90%) [56] is correlated with the presence of both HBsAg and hepatitis B e-antigen (HBeAg), the latter indicating high viral load [57]. Studies conducted on pregnant women and new mothers in Bangladesh [58,59,60,61] indicate an HBeAg rate of 11% to 30% among the HBsAg positive individuals (with the exception of one study with 2 HBsAg positives) (Figure 5). Second, prevalence data among young children (0–4 years old) show a mean prevalence of 3.03% (Figure 6). These two lines of evidence seems to cumulatively imply that vertical transmission is one of the premiere modes of HBV transmission in Bangladesh. What complicates this picture is children in the age group 5–15 consistently show a significantly higher prevalence than under 5 children. This conclusion is based on the findings from four different study populations, differing in region (urban vs. rural) and socioeconomic background [1,3,13,18]. Based on prevalence data alone, while the high mean prevalence among children <15 (5.6%) does indicate the possibility of significant vertical transmission, a closer look at children of different age groups makes this inference less clear-cut.

Two studies have attempted to specifically investigate vertical transmission of HBV in Bangladesh. One study conducted in 1992 reported a vertical transmission rate of 16.66%, with HBeAg positive mothers transmitting in nearly 100% of the cases [61]. A second study, based on sera collected from 330 pairs of new mothers and children (0–8 months old), reached the opposite conclusion [59]. Despite an expected level of prevalence among the new mothers (5.4% in 1993), only 1 of the 334 children showed HBsAg positivity. A third study investigated HBV biomarkers in cord blood [58] but its relevance to our current purposes is limited, as presence of these biomarkers at birth is often transitory and does not necessarily reflect vertical transmission [54].

## 6. Occult Hepatitis B Virus Infection and Implications for Blood Donor Screening 

There are reasons to doubt whether using HBsAg as the exclusive biomarker for HBV infection is adequate, particularly because of the existence of cases where HBsAg negative individuals harbor replicating HBV DNA and even symptoms associated with liver abnormalities. In their 2012 study, Mahtab et al. report 50 cases of HBsAg negative individuals in Bangladesh, 11 of whom had HBV DNA in the 10^2^–10^4^ copies/ml range [62]. However, the study population was chosen based on clinical symptoms ranging from elevated blood alanine aminotransferase (ALT) levels to hepatocellular carcinoma. A more representative analysis was recently produced by Jahan et al., where HBV DNA was detected in 7 out of 398 healthy individuals (1.8%), with five of them having abnormally elevated blood ALT levels (>30 IU/L) [63].

The discussion is particularly concerning if placed in the scaffold of screening of blood donors. Some of the earliest studies on HBV seroprevalence in Bangladesh report very high levels of HBsAg among professional or commercial blood donors (18–29%) [5,6]. Due to the shift from reliance on professional to voluntary blood donors in the last two decades, professional blood donation has ceased to be of great clinical concern in Bangladesh. Voluntary blood donors are declared to be free from HBV infection on the basis of HBsAg screening and usually prevalence among this population group is low as Figure 3 has illustrated. However, given an occult HBV prevalence rate of around 1.8%, supposedly “safe” blood donations can cause transfusion mediated HBV infection. There is very little data as of yet to know the extent of this risk in Bangladesh.

## 7. Molecular Epidemiology

HBV isolates have been classified into 10 genotypes based on their genomic characteristics, with a divergence in excess of 8% constituting a separate genotype [64,65]. Genotype differences between HBV isolates have been implicated in a number of clinical outcomes, including ALT elevation and progression to chronicity [66]. Certain genotypes are also associated with particular routes of transmission, for example—genotypes B and C are associated with high levels of vertical transmission. This link may be somewhat tenuous, however, as vertical transmission is usually associated with areas of high endemicity and genotypes B and C may simply be reflective of the level of endemicity, as opposed to vertical transmission per se [66]. Some studies also report an association between rates of occult infection and genotypes but more recent studies cast doubt on this [67]. Data from India [68] and Pakistan [69] suggest a predominance of genotype D in the subcontinent.

Only four studies have been done on the genotypes of HBV isolates in Bangladesh, while a fifth addresses the issue tangentially based on four samples [22,70,71,72,73]. This amounts to a survey of 160 samples in total, spanning more than a decade. Conclusions on the basis of such limited sample size would need to be drawn provisionally but certain trends nonetheless emerge.

**Figure 7:**
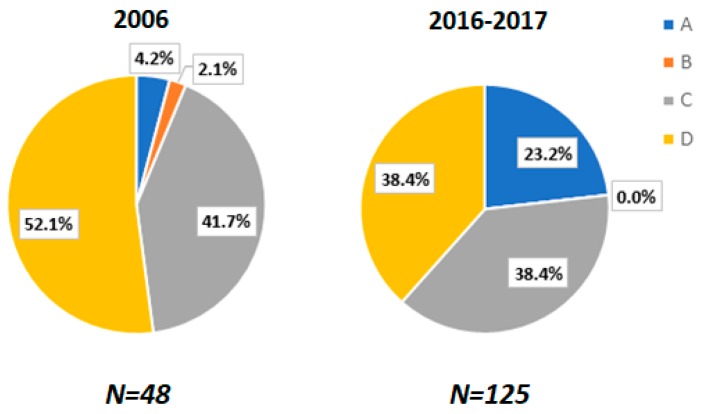
Relative proportions of HBV genotypes in isolates tested in 2006 and 2016–2017.

Of the four major studies, the first one was published in 2006 (sample size 45), with the remaining three being published in 2016–2017 (pooled sample size 115). All of these studies show that genotypes C and D are the most prevalent genotypes in Bangladesh. However, as Figure 7 illustrates, there is a noticeable temporal shift in the pattern of genotype distribution- genotype A has become significantly more common in the newer studies, corresponding to a substantial drop in prominence on the part of genotype D. 

A further caveat to be added is that the majority of the samples studied are from chronic carriers. Only 22 of the isolates are stated as being from acute patients and information is not available about an additional 23 samples. As progression to chronicity have been associated with genotype C and D [66], our analysis may have overestimated the prevalence of these genotypes among the general population.

## 8. Discussion

As seen from our analysis of the studies done on the healthy population groups in Bangladesh, very few of the prevalence rates reported in the studies can be said to accurately represent the general population in Bangladesh, due to post-hoc study design based on convenience sampling. This accentuates the need for more comprehensive epidemiological surveys with larger sample sizes, with the sampling frame chosen to capture regional and sociodemographic disparities. The extant data does indicate, as other authors have pointed out, that HBV infection is in decline in Bangladesh. Identifying the key contributing factors behind this decline, especially in quantitative terms, would require research beyond the scope of this article. Nevertheless, increased vaccination coverage in Bangladesh presents itself as a rather obvious answer. This increase in coverage was brought about by the inclusion of the hepatitis B vaccine in the Expanded Program of Immunization (EPI) in 2003 [74], hailed as one the most successful programs undertaken by the Bangladesh Government [75]. The current coverage exceeds 90% in all 64 districts in Bangladesh, with the overall coverage of the 3rd dose being 90.9% [74]. The WHO-recommended birth dose of the vaccine is yet to be implemented, however, as only a minority of births in Bangladesh (37%, according to most recent estimates) take place in a health facility [74]. In addition, for a disease as stratified across socioeconomic groups as hepatitis B, it is also safe to assume that a key contributing factor behind the decline may have been increased awareness, both of the risk factors and the necessity of vaccination. Again, justifying this assertion would require research beyond our current scope.

There is a near-exclusive reliance on HBsAg to measure prevalence. In addition to being less informative, in that it does not capture the past history of infection, this strategy may also have dangerous implications especially in the context of pre-transfusion blood screening. The strategy in many countries today is to reject anti-HBc positive blood [57]. However, due to the relatively high endemicity of HBV in Bangladesh (recent prevalence rates being as high as 60% in certain risk groups [19], this step would be hardly practical as it would drastically reduce the viable blood donor population. Short of adopting Nucleic Acid Testing (NAT) as a part of the screening program, Jahan et al. recommends excluding anti-HBc positive patients with heightened levels of ALT [63]. Another issue that complicates this picture is the fact that we are still unaware of the risk posed by HBsAg negative blood, as there is precious little data on occult infection rates in Bangladesh.

Studies in Bangladesh have often presented contradictory conclusions about the significance of vertical transmission ([3] for, [1] against). Our survey of the extant literature in Bangladesh showed that data on the topic is indeed very ambiguous and only indirect inferences can be drawn based on prevalence rates. The fact that there has been no recent study (since 1993) which addresses this issue is one of the most significant gaps in HBV research in Bangladesh. Testing of cord blood or serum at or immediately after birth is neither necessary nor relevant for this purpose, as these biomarkers are often transitory during birth. Rather, vertical transmission is established by measuring serum HBsAg at six months in babies born of an infected mother.

Health workers are a particularly important risk group in HBV epidemiology but data on the topic is small in amount and findings of different studies are difficult to reconcile. Technicians and hospital or laboratory assistants who have high occupational exposure to contaminated blood and needle-stick injuries are the most important risk group. Data on individuals specifically involved in the management of chronic HBV patients or its sequelae would be more useful in this regard. A 40-sample study found a rate of 0% prevalence among hemodialysis staff [51], while other studies have reported rates as high as 15%. This indicates the role of work environment, adherence to laboratory safety protocols and the attendant risk of exposure in determining HBV prevalence among the healthcare personnel and data regarding work history and other relevant factors should be collected to aid subsequent interpretation of the results.

Finally, there is a case to be made for a progressively greater incorporation of molecular epidemiology in HBV studies in Bangladesh. Not only is this of general epidemiological importance, as genotypes often determine pathologic potential but also because it overlaps with other research questions the present review has been at pains to address (e.g., vertical transmission and screening of voluntary blood donors). While some data on the distribution of genotypes in Bangladesh has been collected, most of the data is on chronic patients and this may introduce bias in our conclusions. Indeed, a 2017 study finds higher rates of acute infection to be associated with the genotype D. More studies need to be done controlling for the acute/chronic distinction to address this issue.

The present review is the first attempt to synthesize the many disparate strands of HBV epidemiology research in Bangladesh. While noting severe inadequacies in many aspects of the research, we try to piece together the available data to provide concrete but cautious estimates in answer to a number of key epidemiological questions. In brief, our key recommendations for guiding future epidemiology research in Bangladesh are: carefully designed studies on population groups intentionally chosen to represent the general population in Bangladesh; a shift from HBsAg-exclusive screenings, particularly among at risk population groups; more investigations of occult infections, vertical transmission and prevalence among healthcare workers with higher exposure rates; and an increased emphasis on molecular epidemiology.

## Figures and Tables

**Table 1:** Brief description of studies conducted on prevalence among important risk groups of HBV in Bangladesh.

Risk Group	Reference	Study Year	Sample Size	Prevalence	Population/Site Description
Injection Drug users	[37]	1996–1997	129	6.20%	Central Drug Addiction Treatment Center, Dhaka
	[36]	1996–2000	136	19.1%	Mukti, Centre for Treatment and Research of Drug Abuse and Mental Health
	[40]	1999–2000	1236	7.60%	Detoxification Clinics and Needle Exchange Programs in central and northwestern Bangladesh
	[41]	2002	561	9.40%	Needle Exchange program at Dhaka
	[42]	2006–2007	20	25%	Drug addiction treatment clinics in Dhaka
	[43]	2007–2008	145	7.50%	Voluntary health education programs in four border cities in northwestern Bangladesh
	[44]	2012–2013	400	7%	Central Drug Addiction Treatment Center, Dhaka
Commercial Sex workers	[45]	1996	164	9.8%	Dhaka
	[46]	1989	100	11.0%	Dhaka
Thalassemic patients	[47]	2000–2001	152	13.8%	Multitransfused children attending the Dhaka Medical College Hospital (DMCH)
	[48]	2011–2012	100	3.0%	Mutlitransfused children attending the Bangabandhu Sheikh Mujib Medical University (BSMMU)
	[49]	2013	200	6.5%	Multitransfused patients attending the Mymensingh Medical College Hospital (MMCH)
Healthcare workers	[50]	2015	113	7.96%	Doctors, nurses, technicians and assistants at the Chittagong Medical College Hospital
	[51]	2004–2005	40	0%	Hemodialysis staff at the BSMMU
	[52]	2012	80	0%	Physicians at the DMCH
	[53]	1994–1997	210	1.9%	Doctors, nurses and auxiliary staff at the MMCH
